# The Role of Pre-Pandemic Mental Health Status and Personality Traits on Psychological Distress during the COVID-19 Lockdown among Italian Young Adults

**DOI:** 10.3390/bs13020141

**Published:** 2023-02-07

**Authors:** Daniela Marchetti, Lilybeth Fontanesi, Elena Camisasca, Marco Colasanti, Venusia Covelli, Serena Di Giandomenico, Sarah Miragoli, Maria Cristina Verrocchio

**Affiliations:** 1 Department of Psychological, Health and Territorial Sciences, G. d’Annunzio University of Chieti-Pescara, 66100 Chieti, CH, Italy; 2 Faculty of Psychology, E-Campus University, 22060 Novedrate, CO, Italy; 3 Department of Humanities, University of Urbino Carlo Bo, 61029 Urbino, PU, Italy; 4 CRIdee, Trauma Psychology Research Unit, Psychology Department, Catholic University of the Sacred Heart, 20123 Milano, MI, Italy

**Keywords:** psychological distress, anxiety, depression, COVID-19, young adults

## Abstract

The COVID-19 pandemic imposed changes on day-to-day activities and had a detrimental psychological effect on the population, especially among vulnerable individuals, such as adolescents and young adults. The current study aimed to explore variables associated with anxiety, depressive and somatic symptoms in a sample of 608 Italian young adults aged 18 to 25. Data were collected using an online questionnaire administered two months into the COVID-19 lockdown, which explored several areas including sociodemographic information, pre-pandemic and current psychological distress, pre-pandemic and current levels of loneliness, and the traits of intolerance of uncertainty and boredom susceptibility. Results highlighted that having pre-existing mental health issues, being female, and the personality traits of intolerance to uncertainty and boredom susceptibility all played a role in the psychological distress experienced during the pandemic. COVID-19 contributed to negative impacts on young adults’ mental health, highlighting the necessity to develop protective psychological intervention tailored for this vulnerable population.

## 1. Introduction

The coronavirus disease of 2019 (COVID-19) quickly and unexpectedly spread throughout the world, prompting the Italian government to impose a nationwide lockdown starting on 11 March 2020. The subsequent restrictions and protective measures took a toll on the mental health of both general and specific populations (e.g., healthcare workers, parents) [1,2]. Longitudinal studies [3] reported high levels of psychological distress (especially in the dimensions of depression and stress) in the Italian population, with a peak during the final period of the lockdown (May 2020).

The literature indicated that adolescents’ and young adults’ mental health was particularly affected during the period of prolonged social isolation, mainly due to the importance of peer interaction and social bonds for this population. However, it is worth noting that even under normal living conditions, this population is particularly vulnerable: for instance, 20–48% of adolescents and young adults reported severe levels of loneliness [4], with young adults being the group with the highest prevalence of loneliness [5]. 

This population’s vulnerability to mental health problems could be ascribed to its specific evolutionary challenges, such as the transition to adulthood and the hardships associated with beginning autonomous living. In this sense, the pandemic might have hindered the realization of short-term goals associated with the developmental stage, leading young people to experience emotional distress [6]. Indeed, the literature highlighted that the pandemic might have exacerbated the vulnerabilities of specific populations [7], for example, pregnant women [8] and the LGBTQ community [9].

During the COVID-19 pandemic, young adults experienced alarming levels of anxiety, depression, and suicidality, in line with a systematic review that identified younger age (≤40) as a factor associated with distress in the general population [10]. Moreover, a study comparing different age groups during the COVID-19 lockdown revealed that young adults experienced higher levels of emotional distress (depressive and anxiety symptoms) than older age groups [6].

Furthermore, loneliness and alcohol or substance abuse have also been recorded in a large sample of participants aged 18–35 in the United States of America [11]. Padmanabhanunni and Pretorius [12] have also indicated that loneliness is associated with perceived risk of infection, limited knowledge regarding COVID-19 and lower resilience. 

With respect to young adults, the adverse mental health outcomes during the pandemic have been mostly studied in university students. In this specific population, anxiety, depression, and post-traumatic stress symptoms have all been well-documented [13]. In Spain, during the first weeks of lockdown, university students reported high levels of stress, anxiety, and depression [14], compared to university staff members. 

Existing literature on the COVID-19 pandemic’s mental health consequences suggested that state (i.e., a situational and experiential state, given by a circumscribed situation or condition, which might include transitory mental health conditions, such as depressive and anxiety symptoms or psychological distress) and trait factors (i.e., a stable over time trait, mostly affected by genetic factors, such as gender and personality) play important and interactive roles. The literature regarding gender, for instance, consistently indicated being female as a relevant risk factor for the expression of negative emotions and worse mental conditions during the COVID-19 lockdown [1], suggesting the existence of gender differences in vulnerability to stress. 

Among trait factors, one relevant psychological vulnerability factor could be intolerance of uncertainty, which can be conceived as a negative response to ambiguity and is defined as “an individual’s dispositional incapacity to endure the aversive response triggered by the perceived absence of salient, key, or sufficient information, and sustained by the associated perception of uncertainty.” (p. 31) [15]. Intolerance of uncertainty involves the inability to cope with ambiguity and change, which are considered threatening. Higher levels of intolerance of uncertainty have been associated with anxiety-related disorders [16]. There is also substantial evidence connecting intolerance of uncertainty and depression [17]. A systematic review revealed that this trait could be a risk factor for depression and anxiety [18]; its ability to predict a variety of psychopathological symptoms makes it a transdiagnostic vulnerability factor. Even though limited research has investigated the role of intolerance of uncertainty on young adults’ mental health during the COVID-19 pandemic, Glowacz and Schmits [19] documented an increase in anxiety and depression, especially for young adults, partly explained by this variable. Furthermore, intolerance of uncertainty appeared to be associated with higher levels of fear of COVID-19 [20].

Another psychological vulnerability trait-factor that might play a role in young adults’ psychological response to the pandemic and related home confinement is boredom susceptibility, one of the determinants of the experience of boredom along with the characteristics of the environment. Boredom susceptibility is considered the individual tendency to become restless under dull or unchanging conditions and can be defined as “the tendency to be under-aroused because of an impoverished or under-stimulating environment.” (p. 586) [21]. This trait is also regarded as one of the components of sensation seeking, which is the need for varied, new and complex sensations and experiences to maintain a high level of excitement. The literature suggested that boredom susceptibility is closely associated with internalizing symptoms, such as anxiety and depression [22], and individuals who obtained higher scores in the ability to cope with boredom are less likely to experience negative psychological effects [23]. In the context of the COVID-19 pandemic, Boylan et al. [24] highlighted that boredom-prone individuals were less likely to adhere to social isolation and social restriction rules.

To deepen the understanding on how the COVID-19 pandemic affects a population, it is of paramount importance to gather data regarding the population’s prior mental health status, considering that people already affected by a psychological condition might be more vulnerable to stressful situations. Literature indicated that prior depression, anxiety, and somatic symptoms influenced the perceived negativity of subsequent stressors [25] and that individuals with pre-existing mental health conditions were more susceptible to stressors associated with COVID-19 [26]. However, a recent systematic review [27] yielded heterogenous findings, with both improving and deteriorating mental health observed compared to pre-pandemic data, highlighting that the role of pre-existing mental disorders has yet to be determined.

The present study aimed to examine the impact of the COVID-19 pandemic on young adults’ mental health within a cross-sectional research design. Specifically, the role of pre-existing psychological distress (anxiety, depressive, and somatic symptoms) was assessed, with the aim of evaluating whether a previous mental health condition would impact the psychological response during the pandemic. Furthermore, considering that the COVID-19 pandemic represents a situation characterized by high uncertainty, the role of intolerance of uncertainty and boredom susceptibility on psychological distress was assessed. 

The results may deepen the understanding of the role of pre-existing mental conditions, personality traits and gender on the psychological response to the COVID-19 pandemic, assisting government agencies in preserving the psychological wellbeing of a particularly vulnerable population, young adults.

## 2. Materials and Methods

### 2.1. Sample and Procedure

A total of 617 young adults participated in the survey. The inclusion criterion was being aged between 18 and 25 years old. After removing incomplete data, the final sample was composed of 608 Italian young adults (152 males, MAge = 21.99, SD = 2.24; 456 females, MAge = 21.91, SD = 2.22). The main characteristics of the sample are presented in Table 1. After indicating their consent, participants completed an anonymous questionnaire, administered on an online survey platform, Qualtrics. The link to access the survey was disseminated through social networks, including snowball sampling via WhatsApp, to reach a large number of young adults. The survey took approximately 20 min to complete. Participants were informed about the study’s aims, procedures, and data treatment and were told they could interrupt or quit the survey at any point. Data collection started on 9 May 2020, two months into the COVID-19 lockdown in Italy and was completed on 10 June 2020. Participants did not receive any compensation for their participation. The study was approved by the Ethics Committee of the Department of Psychological, Health and Territorial Sciences at the G. d’Annunzio University of Chieti-Pescara (protocol number: 20004).

The survey used for some of its measures a then-test approach by asking participants to complete the survey once thinking about their psychological condition before the COVID-19 lockdown period, and once thinking about their psychological condition during the COVID-19 lockdown.

### 2.2. Measures

The survey covered several areas: (i) sociodemographic information (gender, age, region of residence, and marital, educational, living, and work status); (ii) pre-pandemic and current psychological distress; (iii) pre-pandemic and current levels of loneliness; (iv) intolerance of uncertainty; (v) boredom susceptibility.

#### 2.2.1. Pre-Pandemic and Current Level of Psychological Distress

The Brief Symptom Inventory-18 (BSI-18) [28] was employed to measure pre-pandemic and current psychological distress. It is an 18-item questionnaire used to assess symptoms of psychological distress; each item is scored on a five-point Likert scale (0 = not at all, 1 = a little bit, 2 = moderately, 3 = quite a bit, 4 = extremely). Participants were asked to complete the BSI-18 twice, once according to their recollection of pre-pandemic symptoms and a second time reporting the symptomatology experienced during the past two weeks. The BSI-18 comprises three clinical subscales: somatization (BSI-S), anxiety (BSI-A), and depression (BSI-D), each composed of 6 items. In the current study, the global severity index (GSI-18), which represents an indicator of the overall psychological distress obtained by summing the three subscales, was used. The internal consistency of the BFI-18 pre-pandemic was α = 0.92, while the internal consistency of the BFI-18 during the past two weeks was α = 0.91.

#### 2.2.2. Pre-Pandemic and Current Loneliness

The Three-Item Loneliness Scale (TIL Scale) [29] was administered to measure participants’ perception of social connectedness. This scale is composed of three items, asking participants how often they experience feelings of: (1) lack of companionship; (2) being left out; (3) being isolated from others, on a 3-point Likert scale coded from 1 “hardly ever”, to 3 “often”. The responses were summed up, with higher scores indicating greater loneliness (range 3–9). This scale was administered twice, once according to participants’ recollection of pre-pandemic symptoms and a second time reporting the symptomatology experienced during the past two weeks. In this study, the Cronbach’s alpha indicated good internal consistency (α = 0.78).

#### 2.2.3. Intolerance of Uncertainty

The Intolerance of Uncertainty Scale (IUS-12) [30,31] was employed to measure intolerance of uncertainty, conceptualized as the individual’s tendency to find uncertain situations unpleasant. It is a 12-item short-form of the original 27-item Intolerance of Uncertainty Scale. This scale assesses two factors: prospective IU (7 items, e.g., “I can’t stand being taken by surprise”) and inhibitory IU (5 items, e.g., “When it’s time to act, uncertainty paralyses me”), even though it has been suggested that the general IU factor might have higher utility than the two dimensions separately [32]. Respondents are asked to rate the extent to which each statement applies to themselves on a 5 point-Likert scale (1 = “Not at all like me”, 5 = “Entirely like me”). The internal consistency of the scale was α = 0.83.

#### 2.2.4. Boredom Susceptibility

The Brief Sensation-Seeking Scale (BSSS) [33,34] is an 8-item instrument developed to measure four dimensions of sensation seeking: experience seeking; boredom susceptibility; thrill and adventure seeking; and disinhibition. Each subscale is composed of two items rated on a five-point Likert scale ranging from strongly disagree to strongly agree. For this study, boredom susceptibility was assessed using BSSS’ boredom susceptibility (BS) two items (“I get restless when I spend too much time at home”; “I prefer friends who are excitingly unpredictable”).

#### 2.2.5. Statistical Analysis

Data were analyzed using the Statistical Package for Social Sciences SPSS v.26.0 (IBM SPSS Statistics, New York, NY, USA). Three main steps characterized the data analysis. First, two different groups of young adults, described by their psychological condition (considering loneliness, somatization, depression, and anxiety levels), were identified. Within a person-centered approach, profiles of participants with high and low levels of internalizing symptoms (BSI-S, BSI-D, and BSI-A) and loneliness were identified with cluster analyses based on pre-pandemic scores on the BSI-18 subscales and TIL scale. Study participants were grouped by K-means cluster analysis procedures and standardized mean values of the BSI-18 and loneliness grouping variables describing the characteristics of each identified profile were calculated. In the second step, multivariate analysis of covariance (MANCOVAs) was performed, with clusters as independent variables, and current BSI-18 subscales’ and TIL scale’s scores, IUS-12 score, and BS score as dependent variables. Considering that the present sample was imbalanced in relation to gender, composed of mostly females, gender was entered as a covariate. In the final stage, a stepwise multiple linear regression analysis was conducted to analyze the single contribution of each variable on the psychological condition of young adults during the COVID-19 lockdown. The current GSI-18 was entered as dependent variable. In the first step, clusters were entered as independent variables, followed by gender in the second step, the IUS-12 score in the third step, and lastly, the BS score in the fourth step.

## 3. Results

### 3.1. Cluster Analysis

**Figure 1:**
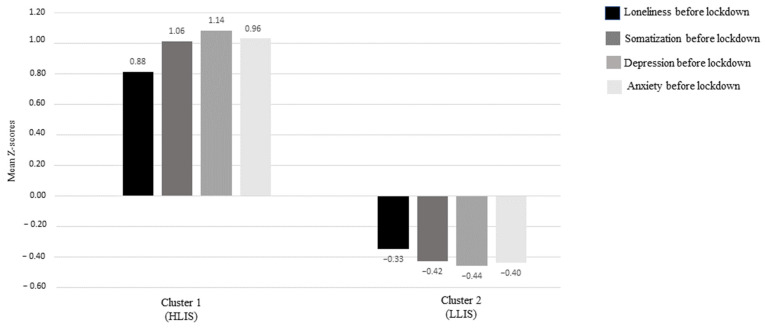
Profiles and z-scores for the clusters.

A k-means cluster analysis was computed to identify two groups. The variables incorporated in the analysis were pre-pandemic internalizing symptoms (i.e., anxiety, depression, and somatization) and levels of loneliness. We looked at a two-group cluster solution. Convergence was achieved after 11 iterations, using a converge criteria of 0.00. The distance between the centers of clusters 1 and 2 was 9.08. The first cluster (High Levels of Internalizing Symptoms (HLIS), *n* = 446, 71.9% female) consisted of participants with high levels (mean z-scores above average) of internalizing symptoms and loneliness, while the second cluster (Low Levels of Internalizing Symptoms (LLIS), *n* = 170, 82.2% female) was composed of individuals with low levels (mean z-scores above average) of internalizing symptoms and loneliness. Profiles and z-scores are reported in Figure 1. The clusters describe two different profiles of young adults participating in the study, based on a self-reported recollection of their psychological condition before the lockdown.

### 3.2. Association between Loneliness and Internalizing Symptoms Profiles and Psychological Condition during the Pandemic

Results from the MANCOVA showed a significant multivariate effect of cluster profiles after controlling for gender, Wilks’ Lambda = 0.71, F (6, 600) = 41.26, *p* < 0.001, *η*^2^ = 0.29. Follow-up multivariate analyses indicated that all the dependent variables differed significantly across profiles, with the exception of boredom susceptibility (see Table 2). Results reported in Table 2 highlighted that the HLIS profile, compared to the LLIS, scored significantly higher in intolerance of uncertainty, levels of loneliness during the pandemic, and levels of internalizing symptoms (anxiety, depression, and somatization) during the pandemic.

### 3.3. Linear Regression Analysis

A four-step linear regression analysis was carried out to evaluate the influence of the levels of internalizing symptoms and loneliness before the lockdown (HLIS and LLIS clusters), gender, intolerance for uncertainty, and boredom susceptibility on the general psychological distress (measured with the GSI-18) during the pandemic (see Table 3). Clusters were entered in the first step and highlighted that belonging to the HLIS cluster accounted for a 26% variance. Gender, added in the second step, added a 3% variance, indicating that females were more at risk of developing internalized symptoms during the lockdown. In the third step, intolerance for uncertainty was added, contributing to a 7% variance. Finally, in the last step, boredom susceptibility was added, contributing for a 3% variance, and the four-step model explained the 39% (R²= 0.388, *p* > 0.001) of the total variance of the GSI-18.

## 4. Discussion

The present study addressed internalizing symptoms and the perception of loneliness in a large sample of Italian young adults aged 18 to 25 during the COVID-19 lockdown, with the purpose of analyzing differences between those who already perceived themselves as lonely and with pre-existing anxiety, depression, and somatic symptoms and those with lower levels of perceived loneliness and internalizing symptoms. Moreover, given the fact that high levels of psychological distress during the pandemic could be associated with stable personality traits (e.g., boredom susceptibility and intolerance of uncertainty) as well as with the pre-pandemic mental health state, the present research aimed to examine the weight of these different variables in influencing psychological distress during the lockdown. 

A large body of literature documented the detrimental effect of COVID-19 on the population’s mental health, especially in vulnerable groups such as young adults [11]. In general, the present study yielded results in line with existing findings and highlighted the negative mental health consequences of the pandemic on the younger population. More in detail, the sample was divided into two groups (HLIS vs. LLIS): one consisted of individuals with high levels of pre-pandemic internalizing symptoms and perceived loneliness and the other included individuals with low levels of pre-pandemic internalizing symptoms and loneliness. The first goal was to explore the difference between the two groups regarding the manifestation of internalizing symptoms, loneliness, boredom susceptibility, and intolerance to uncertainty during the first wave of the COVID-19 pandemic. Results revealed that the two groups differed in all the dependent variables considered except for boredom susceptibility: the HLIS group registered a worsening of their symptomatology, perceived themselves as lonelier than the LLIS group and presented a higher intolerance to uncertainty. In other words, the COVID-19 pandemic and related protective measures seemed to be particularly harmful for those people who already perceived themselves as lonely and who experienced pre-existing anxiety, depressive and somatic symptoms. This is consistent with prior research [35,36] supporting the notion that individuals with pre-existing mental health conditions are more negatively impacted by COVID-19-related stress than those without pre-existing mental health conditions. This is also in line with findings reporting that students with prior depression reported greater increases in anxiety and stress compared to those without pre-existing depressive symptoms [25]. The same can be said for the role of loneliness: our results in this regard are consistent with findings of a longitudinal study that confirmed the association between loneliness and internalizing symptoms during the COVID-19 pandemic [37]. Furthermore, our results seem to support the association between intolerance of uncertainty and anxiety-related disorders [16] and is also consistent with research indicating an increase in psychosomatic complaints during the COVID-19 pandemic associated with intolerance of uncertainty [38].

The present study also aimed to examine the individual effects that different variables exerted on the presentation of internalizing symptoms during the lockdown, distinguishing between state factors (the presence of depressive, anxiety and somatic symptoms and perceived loneliness before the lockdown), and trait factors (intolerance to uncertainty, boredom susceptibility, and gender). Results indicated that a worse mental health outcome during the lockdown, as reflected by the indicator of global psychological distress GSI-18, was mainly explained by high levels of pre-pandemic symptoms of anxiety, depression, and somatization and perceived loneliness, followed by intolerance of uncertainty, female gender and boredom susceptibility. A possible interpretation of this finding is that individuals who perceive themselves as lonelier, depressed, anxious, and having somatic symptoms are more sensitive and apprehensive about their internal states and tend to be more aware of their existing psychological symptoms; this, in turn, could have exacerbated their emotional distress [39]. A particularly interesting result of the present research is the role of intolerance of uncertainty, which confirms the relationship between this variable and mental health during the COVID-19 pandemic, especially in young women [40]. It could be hypothesized that individuals who are less likely to tolerate uncertainty tend to overestimate the risk and the probability of negative outcomes, which in turn might exacerbate the negative impact of stressors. Indeed, people with higher boredom susceptibility, and intolerance to uncertainty might be less able to regulate their emotions when experiencing high levels of stress [41]. Reducing uncertainty, therefore, could be a way to alleviate anxiety, depressive and somatic symptoms [19] and during crises such as the current pandemic, psychological interventions aiming to enhance individuals’ capacity to manage uncertainty could be especially useful [42]. Our results indicated that boredom susceptibility played a role, albeit marginally; this variable had been indicated as a risk factor for stress-related emotional distress experienced during the COVID-19 outbreak [39] and its importance from a practical point of view can not be understated: studies conducted during past epidemics [43] have indicated boredom to be one of the major disincentives to comply with quarantine measures. 

The gender differences in vulnerability to psychological distress, even regarding pandemic-related stressors, are well established [13]. Surprisingly, our results indicated a significant but marginal impact of this variable on GSI-18 scores. Nevertheless, this finding is in line with the existing literature suggesting that females are more likely to develop internalizing symptoms following exposure to stress and trauma [44]; furthermore, females appeared to be particularly vulnerable to mental health problems during the COVID-19 pandemic [45].

These results have many practical implications. First, these findings indicate the importance of preventive interventions on young adults’ mental health before a crisis. It could be useful, in this sense, to screen individuals with pre-existing psychopathological symptoms at an early stage to guarantee timely interventions, with the aim to preserve their physical and psychological fitness [39]. Furthermore, understanding how stressful events are experienced by populations with specific characteristics and vulnerabilities may help identify and improve more suited resources that could be employed in future public health crises. 

Nevertheless, the present study has some limitations. First, the study did not include a pre-pandemic assessment of internalizing symptoms and loneliness, but relied on participants’ recollection, which may have introduced a possible source of bias and/or an overestimation of symptoms; moreover, due to the cross-sectional nature of the study, causality could not be inferred. Furthermore, since the sample was recruited from an online survey, it cannot be considered representative of the Italian young adults’ population. In addition, psychological distress and personality traits were assessed through a self-report instrument, which may have introduced a further source of bias. Finally, there might have been other important variables not included in the present study, such as emotional regulation strategies or possible protective factors (e.g., social support). 

Future studies could attempt to deepen our understanding of how young adults react to stressors and the mechanisms and variables through which these relationships are mediated. The challenges related to this specific population should also be further investigated. It is worth noting that most of the present sample was composed of students (72.9%), who, for instance, might have been particularly affected by the COVID-19 pandemic because of the disruption of their academic activities [46].

In conclusion, these findings highlight how the COVID-19 pandemic contributed to the negative impacts on young adults’ mental health and indicate the necessity to develop tailored protective and preventive psychological interventions towards this vulnerable population.

## Figures and Tables

**Table 1:** Sociodemographic characteristics of the sample.

Variable	*n*	%
**Sex**		
Female	456	75
Male	152	25
**Marital Status**		
Single	275	45.2
In a relationship	280	46.1
Married/Cohabitant	15	2.5
Other	38	6.3
**Educational Level**		
Middle school diploma	53	8.7
High school diploma	363	59.7
Bachelor’s degree	122	20.1
Master’s degree	61	10.0
Post-Lauream	9	1.5
**Work Status**		
Freelancer	11	1.8
Employee	53	8.7
Unemployed	50	8.2
Student	443	72.9
Other	51	8.4
**Region of Residence**		
North	184	30.4
Center	76	15.0
South	348	54.6
**Lockdown Living Condition**		
Alone	20	3.3
With Roommates	33	5.4
With Family	508	83.6
With Partner	41	6.7
Other	6	1.0

**Table 2:** Multivariate analyses of covariance, between LLIS and HLIS clusters, in the study variables during the lockdown.

Variable	HLIS ^1^ (*n* = 180)	LLIS ^2^ (*n* = 428)	*F*	*η*²
IUS-12 ^3^	4.92	35.46	45.73 ***	0.131
BS ^4^	6.03	5.91	1.43	0.005
Loneliness	6.84	5.58	36.96 ***	0.109
BSI-S ^5^	6.09	2.30	89.54 ***	0.228
BSI-D ^6^	11.4	6.41	82.87 ***	0.215
BSI-A ^7^	6.59	8.04	82.17 ***	0.215

Note. All the independent variables refer to the period during the lockdown. ^1^ HLIS: High levels of internalizing symptoms and loneliness cluster. ^2^ LLIS: Low levels of internalizing symptoms and loneliness cluster. ^3^ IUS: Intolerance of uncertainty. ^4^ BS: Boredom susceptibility. ^5^ BSI-S: Brief Symptom Inventory-18 Somatization scale. ^6^ BSI-D: Brief Symptom Inventory-18 Depression scale. ^7^ Brief Symptom Inventory-18 Anxiety scale. *** *p* < 0.001.

**Table 3:** Stepwise multiple linear regression analysis, with GSI-18 Index during the lockdown as a dependent variable.

Variable	Step 1			Step 2			Step 3			Step 4		
	*B*	*SEB*	*β*	*B*	*SEB*	*β*	*B*	*SEB*	*β*	*B*	*SEB*	*β*
Cluster	−13.7	0.94	−0.51	−13.18	0.93	−0.49	−1.7	0.93	−0.4	−1.6	0.91	−0.39
Gender	-	-	-	4.92	0.98	0.17	3.7	0.94	0.131	4.01	0.92	0.14
IUS-12 ^1^	-	-	-	-	-	-	0.48	0.06	0.29	0.47	0.06	0.29
BS ^2^	-	-	-	-	-	-	-	-	-	1.17	0.23	0.16
*R*	0.509			0.537			0.602			0.623		
*R*²	0.259			0.289			0.363			0.388		
Δ*R*²	0.259			0.03			0.074			0.026		

Note. Cluster: LLIS = 1; HLIS = 2; Gender: Male = 1; Female = 2. ^1^ IUS: Intolerance of uncertainty. ^2^ BS: Boredom susceptibility. All the betas were significant for *p* < 0.001.

## Data Availability

The data presented in this study are available from the corresponding author upon reasonable request.

## References

[1] Marchetti D., Fontanesi L., Mazza C., Di Giandomenico S., Roma P., Verrocchio M.C. (2020). Parenting-Related Exhaustion during the Italian COVID-19 Lockdown. J. Pediatr. Psychol..

[2] Conti C., Fontanesi L., Lanzara R., Rosa I., Porcelli P. (2020). Fragile Heroes. The Psychological Impact of the COVID-19 Pandemic on Health-Care Workers in Italy. PLoS ONE.

[3] Mazza C., Ricci E., Colasanti M., Cardinale A., Bosco F., Biondi S., Tambelli R., Di Domenico A., Verrocchio M.C., Roma P. (2022). How Has COVID-19 Affected Mental Health and Lifestyle Behaviors after 2 Years? The Third Step of a Longitudinal Study of Italian Citizens. Int. J. Environ. Res. Public Health.

[4] Williams S.E., Braun B. (2019). Loneliness and Social Isolation—A Private Problem, A Public Issue. J. Fam. Consum. Sci..

[5] Cigna International. Cigna U.S (2018). Loneliness Index.

[6] Gambin M., Sękowski M., Woźniak-Prus M., Wnuk A., Oleksy T., Cudo A., Hansen K., Huflejt-Łukasik M., Kubicka K., Łyś A.E. (2021). Generalized Anxiety and Depressive Symptoms in Various Age Groups during the COVID-19 Lockdown in Poland. Specific Predictors and Differences in Symptoms Severity. Compr. Psychiatry.

[7] Nam S.H., Nam J.H., Kwon C.Y. (2021). Comparison of the mental health impact of COVID-19 on vulnerable and non-vulnerable groups: A systematic review and meta-analysis of observational studies. Int. J. Environ. Res. Public Health.

[8] Craig F., Gioia M.C., Muggeo V., Cajiao J., Aloi A., Martino I., Tenuta F., Cerasa A., Costabile A. (2021). Effects of maternal psychological distress and perception of COVID-19 on prenatal attachment in a large sample of Italian pregnant women. J. Affect. Disord..

[9] Salerno J.P., Williams N.D., Gattamorta K.A. (2020). LGBTQ populations: Psychologically vulnerable communities in the COVID-19 pandemic. Psychol. Trauma.

[10] Xiong J., Lipsitz O., Nasri F., Lui L.M.W., Gill H., Phan L., Chen-Li D., Iacobucci M., Ho R., Majeed A. (2020). Impact of COVID-19 Pandemic on Mental Health in the General Population: A Systematic Review. J. Affect. Disord..

[11] Horigian V.E., Schmidt R.D., Feaster D.J. (2021). Loneliness, Mental Health, and Substance Use among US Young Adults during COVID-19. J. Psychoact. Drugs.

[12] Padmanabhanunni A., Pretorius T.B. (2021). The Unbearable Loneliness of COVID-19: COVID-19-Related Correlates of Loneliness in South Africa in Young Adults. Psychiatry Res..

[13] Hawes M.T., Szenczy A.K., Klein D.N., Hajcak G., Nelson B.D. (2022). Increases in Depression and Anxiety Symptoms in Adolescents and Young Adults during the COVID-19 Pandemic. Psychol. Med..

[14] Odriozola-González P., Planchuelo-Gómez Á., Irurtia M.J., de Luis-García R. (2020). Psychological Effects of the COVID-19 Outbreak and Lockdown among Students and Workers of a Spanish University. Psychiatry Res..

[15] Carleton R.N. (2016). Into the Unknown: A Review and Synthesis of Contemporary Models Involving Uncertainty. J. Anxiety Disord..

[16] Rosser B.A. (2019). Intolerance of Uncertainty as a Transdiagnostic Mechanism of Psychological Difficulties: A Systematic Review of Evidence Pertaining to Causality and Temporal Precedence. Cogn. Ther. Res..

[17] Boelen P.A., Vrinssen I., van Tulder F. (2010). Intolerance of Uncertainty in Adolescents: Correlations with Worry, Social Anxiety, and Depression. J. Nerv. Ment. Dis..

[18] Gentes E.L., Ruscio A.M. (2011). A Meta-Analysis of the Relation of Intolerance of Uncertainty to Symptoms of Generalized Anxiety Disorder, Major Depressive Disorder, and Obsessive–Compulsive Disorder. Clin. Psychol. Rev..

[19] Glowacz F., Schmits E. (2020). Psychological Distress during the COVID-19 Lockdown: The Young Adults Most at Risk. Psychiatry Res..

[20] Mertens G., Gerritsen L., Duijndam S., Salemink E., Engelhard I.M. (2020). Fear of the Coronavirus (COVID-19): Predictors in an Online Study Conducted in March 2020. J. Anxiety Disord..

[21] Mercer-Lynn K.B., Flora D.B., Fahlman S.A., Eastwood J.D. (2013). The Measurement of Boredom: Differences Between Existing Self-Report Scales. Assessment.

[22] Spaeth M., Weichold K., Silbereisen R.K. (2015). The Development of Leisure Boredom in Early Adolescence: Predictors and Longitudinal Associations with Delinquency and Depression. Dev. Psychol..

[23] Boschloo L., Vogelzangs N., van den Brink W., Smit J.H., Beekman A.T.F., Penninx B.W.J.H. (2013). The Role of Negative Emotionality and Impulsivity in Depressive/Anxiety Disorders and Alcohol Dependence. Psychol. Med..

[24] Boylan J., Seli P., Scholer A.A., Danckert J. (2021). Boredom in the COVID-19 Pandemic: Trait Boredom Proneness, the Desire to Act, and Rule-Breaking. Pers. Individ. Dif..

[25] Husky M.M., Kovess-Masfety V., Gobin-Bourdet C., Swendsen J. (2021). Prior Depression Predicts Greater Stress during COVID-19 Mandatory Lockdown among College Students in France. Compr. Psychiatry.

[26] Asmundson G.J.G., Paluszek M.M., Landry C.A., Rachor G.S., McKay D., Taylor S. (2020). Do Pre-Existing Anxiety-Related and Mood Disorders Differentially Impact COVID-19 Stress Responses and Coping?. J. Anxiety Disord..

[27] Kunzler A.M., Röthke N., Günthner L., Stoffers-Winterling J., Tüscher O., Coenen M., Rehfuess E., Schwarzer G., Binder H., Schmucker C. (2021). Mental burden and its risk and protective factors during the early phase of the SARS-CoV-2 pandemic: Systematic review and meta-analyses. Glob. Health.

[28] Derogatis L.R. (1975). Brief Symptom Inventory.

[29] Hughes M.E., Waite L.J., Hawkley L.C., Cacioppo J.T. (2004). A Short Scale for Measuring Loneliness in Large Surveys: Results From Two Population-Based Studies. Res. Aging.

[30] Carleton R.N., Norton M.A.P.J., Asmundson G.J.G. (2007). Fearing the Unknown: A Short Version of the Intolerance of Uncertainty Scale. J. Anxiety Disord..

[31] Bottesi G., Ghisi M., Novara C., Bertocchi J., Boido M., De Dominicis I., Freeston M. (2015). Intolerance of Uncertainty Scale (IUS-27 e IUS-12): Due studi preliminari. Psicoter. Cogn. Comport..

[32] Bottesi G., Noventa S., Freeston M.H., Ghisi M. (2019). Seeking Certainty about Intolerance of Uncertainty: Addressing Old and New Issues through the Intolerance of Uncertainty Scale-Revised. PLoS ONE.

[33] Hoyle R.H., Stephenson M.T., Palmgreen P., Lorch E.P., Donohew R.L. (2002). Reliability and Validity of a Brief Measure of Sensation Seeking. Pers. Individ. Dif..

[34] Primi C., Narducci R., Benedetti D., Donati M.A., Chiesi F. (2011). Validity and reliability of the Italian version of the Brief Sensation Seeking Scale (BSSS) and its invariance across age and gender. TPM Test. Psychom. Methodol. Appl. Psychol..

[35] Castellini G., Rossi E., Cassioli E., Sanfilippo G., Innocenti M., Gironi V., Silvestri C., Voller F., Ricca V. (2021). A Longitudinal Observation of General Psychopathology before the COVID-19 Outbreak and during Lockdown in Italy. J. Psychosom. Res..

[36] Murphy L., Markey K., O’Donnell C., Moloney M., Doody O. (2021). The Impact of the COVID-19 Pandemic and Its Related Restrictions on People with Pre-Existent Mental Health Conditions: A Scoping Review. Arch. Psychiatr. Nurs..

[37] Velotti P., Rogier G., Beomonte Zobel S., Castellano R., Tambelli R. (2021). Loneliness, Emotion Dysregulation, and Internalizing Symptoms During Coronavirus Disease 2019: A Structural Equation Modeling Approach. Front. Psychiatry.

[38] Gica S., Kavakli M., Durduran Y., Ak M. (2020). The effect of COVID-19 pandemic on psychosomatic complaints and investigation of the mediating role of intolerance to uncertainty, biological rhythm changes and perceived COVID-19 threat in this relationship: A web-based community survey. Personnel.

[39] Yan L., Gan Y., Ding X., Wu J., Duan H. (2021). The Relationship between Perceived Stress and Emotional Distress during the COVID-19 Outbreak: Effects of Boredom Proneness and Coping Style. J. Affect. Disord..

[40] Del Valle M.V., Andrés M.L., Urquijo S., Yerro-Avincetto M., López-Morales H., Canet-Juric L. (2020). Intolerance of Uncertainty over COVID-19 Pandemic and Its Effect on Anxiety and Depressive Symptoms. Rev. Interam. Psicol./Interam. J. Psychol..

[41] Culp N.A. (2006). The Relations of Two Facets of Boredom Proneness with the Major Dimensions of Personality. Pers. Individ. Dif..

[42] Voitsidis P., Nikopoulou V.A., Holeva V., Parlapani E., Sereslis K., Tsipropoulou V., Karamouzi P., Giazkoulidou A., Tsopaneli N., Diakogiannis I. (2021). The Mediating Role of Fear of COVID-19 in the Relationship between Intolerance of Uncertainty and Depression. Psychol. Psychother. Theory Res. Pract..

[43] DiGiovanni C., Conley J., Chiu D., Zaborski J. (2004). Factors Influencing Compliance with Quarantine in Toronto During the 2003 SARS Outbreak. Biosecur. Bioterror..

[44] Tolin D.F., Foa E.B. (2008). Sex Differences in Trauma and Posttraumatic Stress Disorder: A Quantitative Review of 25 Years of Research. Psychol. Trauma.

[45] Rodriguez-Besteiro S., Tornero-Aguilera J.F., Fernández-Lucas J., Clemente-Suárez V.J. (2021). Gender Differences in the COVID-19 Pandemic Risk Perception, Psychology, and Behaviors of Spanish University Students. Int. J. Environ. Res. Public Health.

[46] Sirotkin A.V., Pavlíková M., Hlad Ľ., Králik R., Zarnadze I., Zarnadze S., Petrikovičová L. (2023). Impact of COVID-19 on University Activities: Comparison of Experiences from Slovakia and Georgia. Sustainability.

